# Contact with nature for emotion regulation: the roles of nature connectedness and beauty engagement in urban young adults

**DOI:** 10.1038/s41598-023-48756-4

**Published:** 2023-12-04

**Authors:** Xuan Gu, Hailin Zheng, Chi-Shing Tse

**Affiliations:** 1 Department of Social Work, Shandong University, Jinan, China; 2 Center for Animal Protection Studies, Shandong University, Jinan, China; 3 Department of Applied Psychology, Guangdong University of Finance and Economics, Guangzhou, China; 4 Department of Educational Psychology, The Chinese University of Hong Kong, Hong Kong, China; 5 Centre for Learning Sciences and Technology, The Chinese University of Hong Kong, Hong Kong, China

**Keywords:** Psychology, Human behaviour

## Abstract

Contact with nature has emotional benefits, but the psychological mechanism and potential moderator underlying the association between nature contact and emotion regulation remain unclear. The present study investigated how self-reported frequency of nature contact is associated with the use of emotion regulation strategies and explored the mediating role of nature connectedness (i.e., psychological connection to nature) and the moderating role of engagement with natural beauty. Employing mediation and moderated mediation analyses, in a cross-sectional sample of 2097 young adults aged 18–35 years old (*M* = 24.01, *SD* = 4.80) residing in urban China, we obtained three major findings. First, nature connectedness mediated the associations between direct/indirect nature contact and cognitive reappraisal as well as expressive suppression. Second, engagement with natural beauty moderated the path from direct/indirect nature contact to cognitive reappraisal in the mediation models. Third, engagement with natural beauty moderated the path from indirect nature contact to nature connectedness in the mediation models. Our study is the first to reveal mediating and moderating factors in the relationships among direct/indirect contact with nature, nature connectedness, engagement with natural beauty, and emotion regulation strategies. These findings provide support for the emotional health of nature contact and have implications for nature-based education and urban planning.

## Introduction

The process of urbanization is advancing at a rapid pace on a global scale, with 55% of the world’s population now living in urban areas, a proportion that is expected to increase to 68% by 2050^1^. This transition from rural to urban lifestyles is accompanied by a notable reduction in individuals’ exposure to natural environments^2^, and a burgeoning trend among young adults to embrace a leisure lifestyle about “getting out in nature”^3,4^. Concurrently, the rise of urbanization coincides with evidence that young adults often face multiple stressors and emotional distress, but they suffer from a weak ability to regulate emotions^5,6^. In light of these considerations, we aim to investigate whether and how contact with nature in young adults serves as a tool to enhance their emotion regulation strategies.

### Nature contact and emotion regulation

Exposure to or contact with non-threatening natural environments is positively associated with improved health and well-being^7–9^. Among the benefits associated with nature contact, we consider the affective ones^10–12^ and particularly examine the moderators and mediators at the individual level. The forms of nature contact include, for instance, being physically present in forest^13^ or indoor space with vegetation^14^ and immersion in virtual natural environments^15^. These various forms of contact with nature can be categorized into the unintentional (e.g., exposure to greenspaces in the neighborhood), direct (e.g., visiting natural spaces), and indirect (e.g., through television programs)^16^. However, the connections between indirect or direct contact with nature and emotion regulation strategies, and the psychological mechanisms underlying the associations, remain relatively less established. We aim to test directly the associations between nature contact and emotion regulation strategies and the mechanism and boundary conditions of these effects.

Emotion regulation refers to processes that influence which emotions people have, when people have the emotions, and how people experience or express these emotions^17^. While studies have identified ways to regulate emotions, cognitive reappraisal and expressive suppression are the most studied emotion regulation strategies^18^. Cognitive reappraisal involves cognitive changes that reframe potentially emotion-eliciting situations in ways that alter their emotional impact; in contrast, expressive suppression involves inhibiting ongoing emotional expressions^17^. Cognitive reappraisal, rather than expressive suppression, is associated with increased positive well-being outcomes and better interpersonal functioning^19^. Adaptive strategies of cognitive emotion regulation are negatively associated with fears and social anxiety^20^. Past studies also found that expressive suppression plays a role in the development and maintenance of psychopathology. For example, expressive suppression was positively associated with depression and anxiety at present^21^ and later in life^22^. Thus, cognitive reappraisal acts as an adaptive emotion regulation strategy while expressive suppression acts as a maladaptive emotion regulation strategy. In the present study, we examined the possible role of nature contact on these two emotion regulation strategies in young adults and explored the psychological mechanism underlying these associations.

### The mediating role of nature connectedness

Adults’ contact with nature can improve their feelings or cognition of connectedness to the natural world^23^. Nature contact mainly refers to the interaction with real or virtual nature, while nature connectedness represents a psychological connection between individuals and nature expressed at the emotional and cognitive levels^24^. Nature connectedness is commonly viewed as a stable trait^25,26^, but it is noteworthy that this trait can be influenced^27,28^, such as demonstrated by studies where exposure to nature-related videos or engaging in walks within natural surroundings led to temporary changes in nature connectedness^29^.

The extent to which people are connected to nature varies. This connectedness, emerging as an important psychological construct for a sustainable future^30^, benefits both human and planetary health and sustainability. People with higher nature connectedness reported to do more pro-environmental behaviors^31,32^, as well as exhibited higher levels of well-being^33,34^. Moreover, nature connectedness predicted young adults’ adaptive vs. maladaptive cognitive strategies of emotion regulation, i.e., reappraisal vs. rumination, which in turn predicted a decline in their perceived stress^35^.

Nonetheless, prior research overlooked the potential role of nature connectedness as a mediating mechanism in the association between nature contact and cognitive reappraisal or expressive suppression strategy of emotion regulation. Therefore, we predict that:

**Hypothesis 1a:** *Nature connectedness positively mediates the relationship between nature contact and cognitive reappraisal strategy of emotion regulation.*

**Hypothesis 1b:** *Nature connectedness negatively mediates the relationship between nature contact and expressive suppression strategy of emotion regulation.*

### The moderating role of engagement with natural beauty

Although nature contact may be associated with emotion regulation strategies through the mediating role of nature connectedness, not all individuals living a lifestyle of frequent contact with nature increase their nature connectedness or use of adaptive emotion regulation strategy. Thus, it is important to explore those factors that may increase or diminish (i.e., moderate) the strength of the associations among nature contact, nature connectedness, and emotion regulation strategies.

The tendency to appreciate natural beauty when one is exposed to or interacts with the natural environment may play a key role. Engagement with natural beauty refers to the general disposition to perceive beauty, gain aesthetic experience, and be touched by the beauty of the natural world^36,37^. Individuals with higher engagement with natural beauty reported higher levels of well-being^38^ as well as concern for the well-being of others^39^ than those with lower engagement. Additionally, there exists correlations between perception of natural beauty and personality traits, which are closely associated with well-being such as openness and gratitude^38^. These findings indicate that individuals who are inclined to appreciate the beauty of nature tend to report higher levels of well-being.

A few studies have highlighted the significance of engagement with natural beauty as a crucial factor contributing to the well-being benefits associated with nature connectedness or nature contact. Frequent contact with nature often evokes a positive appraisal of its beauty^40^. In a thematic analysis of a personal journey, researchers revealed that nature connectedness was a manifestation of personal fulfillment in the landscape, notably through an engagement with the beauty of nature^41^. Moreover, three studies demonstrated different triadic relationships among engagement with natural beauty, nature connectedness, and well-being. Capaldi et al.^42^ found that nature connectedness mediated the relationship between engagement with natural beauty and well-being. Richardson and McEwan^43^ found that engagement with natural beauty mediated the relationship between nature connectedness and well-being, whereas Zhang, Howell, and Iyer^44^ showed that engagement with natural beauty moderated the relationship between nature connectedness and well-being.

Despite these insights, however, to our knowledge, no previous research examined the moderating effect of engagement with natural beauty in the direct and indirect relationships between nature contact and emotion regulation strategies. Given that previous findings of engagement with natural beauty have been mixed, without presupposing a specific moderating pattern, we, therefore, hypothesized that:

**Hypothesis 2:** *Engagement with natural beauty moderates the direct and indirect links between nature contact and emotion regulation strategies.*

**Figure 1:**
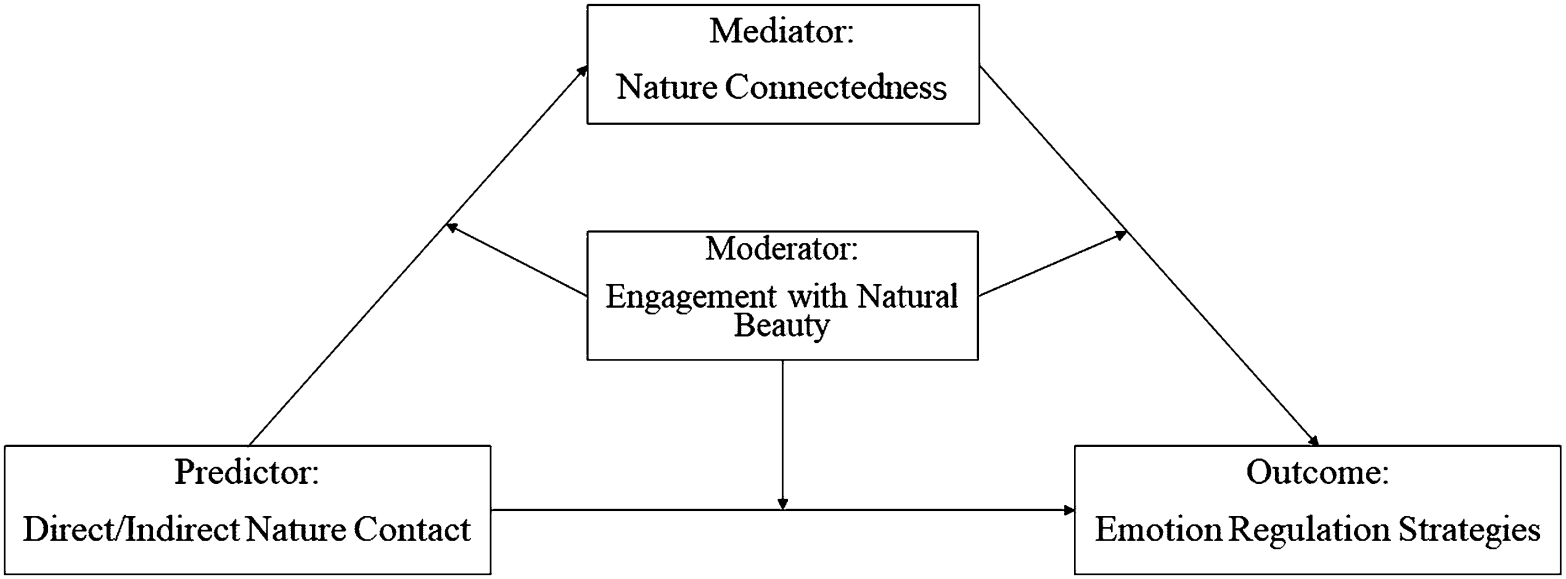
The proposed moderated mediation model.

In summary, we present a theoretical model, illustrated in Fig. 1, to investigate the interrelationships among young adults’ contact with nature, engagement with natural beauty, nature connectedness, and emotion regulation strategies. We conducted a cross-sectional study on young adults who resided in urban China. Developing countries, such as China, have gradually come to realize that the rapid urbanization in the early stages has had adverse effects on ecological quality, and they actively seek the health and sustainable development of cities. Researching the natural environment in Chinese cities for its benefits to residents’ health not only contributes valuable insights and recommendations to China’s green development policies but also provides valuable reference points for the global trend of urbanization. Although we can only infer an association instead of causality from cross-sectional data, researchers can use the data to develop hypotheses about causal relationships^45^.

## Methods

### Participants

Young adults aged 18–35 years who resided in urban areas of mainland China were recruited as participants through various channels, including university courses, psychological health-training programs, and WenJuanXing’s sample services, a popular online survey platform in China that meets scientific standards. The regional distribution of participants, covered all provinces, autonomous regions, and municipalities in Mainland China, except for Qinghai and Xizang.

The investigation was approved by the University Ethics Committee at Guangdong University of Finance and Economics. In accordance with Chinese national policy, as this study used questionnaires and did not collect or disclose any data related to scientific research on human life sciences and medical issues, ethical approval documents were not required. Our study followed the Declaration of Helsinki and the ethical guidelines for research involving human participants. Prior to participation, all participants provided informed consent. They were informed that their participation was voluntary and that their privacy would be protected. In addition, they could contact the first and second authors if they had any questions/concerns.

To ensure data validity, we included in the questionnaire seriousness check questions (e.g., “Please select ‘disagree’ for this question)^46^. We excluded participants who failed to pass the checking or completed the questionnaire in less than 100 s. A total of 2579 participants responded. The final sample comprised 2097 participants. There were 899 males and 1198 females, with a mean age of 24.01 (*SD* = 4.80) years old. Thirty-seven participants obtained a high school degree or below, 696 obtained a college or undergraduate degree, 1195 were current undergraduate students, and 169 obtained a master’s degree or above. A sensitivity power analysis using G*Power version 3.1^47^ revealed that the final sample size allowed us to detect a markedly small effect size (Cohen’s *f*^2^ = 0.005) at an alpha level of 0.05 and statistical power of 95% for the dependent variables in our models.

### Measures

The four scales below were distributed in a fixed order with each scale presenting on a separate section of the online questionnaire.

#### Nature contact

Following previous research^16^, two indices of nature contact were operationalized, i.e., direct and indirect nature contact. Participants responded to the questions on a 5-point scale (1 = never, 5 = always), “How often do you visit nature (e.g., parks, gardens, forest land, etc.) to do activities or exercise?” and “How often do you watch or listen to nature programs (e.g., documentaries, radio, videos, movies, etc.)?”. As the correlation coefficient between direct and indirect nature contact was 0.27 (*p* < 0.01), we separately analyzed the two variables. The higher the score, the higher the frequency of contact with nature.

#### Connectedness to nature

Mayer and Frantz^25^ developed the Connectedness to Nature Scale to measure an individual’s level of connection to the natural world. On a 5-point scale (1 = strongly disagree, 5 = strongly agree), participants responded to each of the 14 items, such as “I recognize and appreciate the intelligence of other living organisms.” A higher score means a person is more connected to nature. This scale has shown good reliability and validity among Chinese participants^48^. Cronbach’s *α* was 0.70 in the study.

#### Emotion regulation strategies

We assessed individual differences in emotion regulation strategies with the 10-item Emotion Regulation Questionnaire^19^. The questionnaire contains two dimensions: Cognitive reappraisal and expressive suppression. Example items are “I control my emotions by changing the way I think about the situation I’m in” and “I control my emotions by not expressing them.” Participants indicated how much they agreed with each item on a 7-point scale (1 = strongly disagree, 7 = strongly agree), with higher scores indicating more tendency to use cognitive reappraisal or expressive suppression strategy. The Chinese version of this scale has shown good reliability and validity^49^. Cronbach’s* α*s of the two dimensions in the study were 0.77 and 0.82, respectively.

#### Tendency to perceive natural beauty

The 4-item Engagement with Natural Beauty Scale^36^ measures an individual’s tendency to perceive and experience natural beauty (e.g., “When perceiving beauty in nature I feel changes in my body, such as a lump in my throat, an expansion in my chest, faster heartbeat, or other bodily responses.”) on a 7-point scale (1 = strongly disagree; 7 = strongly agree). We translated the scale to Chinese by the back-translation method. A higher score means a person is more likely to perceive and experience natural beauty. Cronbach’s *α* in the study was 0.69.

### Data analyses

Standard parametric assumptions were tested. First, whether the data followed normal distribution was examined. The skewness and kurtosis of all variables fell within the acceptable range (i.e., skewness <|2.0| and kurtosis <|7.0|). Second, multicollinearity was not a serious threat according to the variance inflation factors (VIF < 5).

To mitigate the potential issue of common method bias in self-reported data, we conducted Harman’s one-factor test on all measurement items. The results indicated that the first factor accounted for only a small proportion of the variance (18.63% < 40%). There was no general factor in the unrotated factor structure. Therefore, common method variance is unlikely to pose a significant threat in the present study.

We applied Model 4 of the SPSS macro PROCESS^50^ to examine the mediating effects of nature connectedness on the relationships between nature contact and the emotion regulation strategies. We applied Model 59 of the SPSS macro PROCESS to test the moderated mediation model, with the moderating effects of engagement with natural beauty in the direct and indirect links between nature contact and nature connectedness. The bootstrap confidence intervals (CIs) determine whether the effects in Models 4 and 59 are significant based on 5000 random samples. An effect is regarded as significant if the CI does not include zero.

Demographic variables, including gender, age, and education level, were controlled as covariates. Gender and education level were recorded as dummy variables with female and undergraduate students as the reference groups (values = 0), respectively.

## Results

### Correlations between variables

Table 1 displays the means, standard deviations, and Pearson correlation coefficients between the variables. Young adults’ direct nature contact was positively correlated with cognitive reappraisal, connectedness to nature, and engagement with natural beauty. However, direct nature contact was not correlated with expressive suppression. Indirect nature contact was positively correlated with cognitive reappraisal and engagement with natural beauty, while it was not correlated with connectedness to nature or expressive suppression.

**Table 1:** Descriptive statistics and correlations between study variables (*N* = 2097).

	*M*	*SD*	1	2	3	4	5	6
1. Direct nature contact	2.40	1.11	1					
2. Indirect nature contact	2.27	1.12	0.27**	1				
3. Connectedness to nature	4.05	0.41	0.10**	0.03	1			
4. Cognitive reappraisal	5.37	0.77	0.15**	0.11**	0.40**	1		
5. Expressive suppression	4.06	1.39	− 0.04	− 0.04	− 0.12**	− 0.02	1	
6. Engagement with natural beauty	5.32	0.88	0.15**	0.12**	0.43**	0.45**	0.01	1

**p < .01, *p < .05.

### Testing for mediation effects between direct nature contact and emotion regulation strategies

The two emotion regulation strategies, i.e., cognitive reappraisal and expressive suppression, were treated as separate outcomes in all models. Table 2 presents the results of the mediation analyses with direct nature contact as the predictor of mediation model. First, after controlling for covariates, as shown in Model 1a, the results revealed that direct nature contact was positively associated with cognitive reappraisal, *β* = 0.102, SE = 0.015, *p* < 0.001, 95% CI: [0.071,0.132]. Second, as seen in Model 2, the results showed that direct nature contact was positively associated with nature connectedness, *β* = 0.042, SE = 0.008, *p* < 0.001, 95% CI: [0.026, 0.058]. Third, as seen in Model 3a, the association between direct contact nature and cognitive reappraisal was significant, *β* = 0.070, SE = 0.014, *p* < 0.001, 95% CI: [0.042, 0.098]; and the association between nature connectedness and cognitive reappraisal was significant as well, *β* = 0.752, SE = 0.037, *p* < 0.001, 95% CI: [0.679, 0.826]. The findings suggested that nature connectedness partially mediated the relationship between direct nature contact and cognitive reappraisal (indirect effect = 0.032, SE = 0.007, 95% CI: [0.019, 0.045]). The mediation effects account for 31.37% of the total effect of direct nature contact on cognitive reappraisal.

**Table 2:** Testing the mediation effects of nature connectedness with direct nature contact as the predictor.

Predictors	Model 1a (Cognitive reappraisal)		Model 1b (Expressive suppression)		Model 2 (Nature connectedness)		Model 3a (Cognitive reappraisal)		Model 3b (Expressive suppression)	
	*β*	*t*	*β*	*t*	*β*	*t*	*β*	*t*	*β*	*t*
Gender (ref. female)	0.010	0.288	0.213	3.476**	− 0.088	− 4.807***	0.076	2.411*	0.180	2.934**
Age	0.000	− 0.067	− 0.040	− 3.651***	− 0.002	− 0.613	0.001	0.196	− 0.041	− 3.743***
Education level (ref. undergraduate students)										
High school degree or below	0.219	1.629	0.392	1.625	− 0.086	− 1.201	0.284	2.305*	0.359	1.498
Undergraduate or college degree	0.064	1.018	0.083	0.733	0.003	0.076	0.062	1.078	0.083	0.746
Master’s degree or above	0.013	0.178	− 0.209	− 1.637	− 0.039	− 1.028	0.042	0.646	− 0.224	− 1.764
Direct contact	0.102	6.555***	− 0.034	− 1.227	0.042	5.097***	0.070	4.890***	− 0.018	− 0.650
Nature connectedness							0.752	20.066***	− 0.380	− 5.207***
*R*^*2*^	0.026		0.023		0.023		0.183		0.036	
*F*	9.216***		8.320***		8.286***		66.938***		11.093***	

***p < .001, **p < .01, *p < .05; Models 1a, 2, and 3a show the mediating effect of nature connectedness between direct contact and cognitive reappraisal; Models 1b, 2, and 3b show the mediating effect of nature connectedness between direct contact and expressive suppression.

As shown in Model 1b of Table 2, direct nature contact was not associated with expressive suppression, *β* = − 0.034, SE = 0.028, *p* = 0.220, 95% CI: [− 0.089, 0.020]. As seen in Model 3b, the association between direct contact and expressive suppression was not significant, *β* = − 0.018, SE = 0.028, *p* = 0.516, 95% CI: [− 0.073, 0.036]; but the association between nature connectedness and expressive suppression was significant, *β* = − 0.380, SE = 0.073, *p* < 0.001, 95% CI: [− 0.523, − 0.237]. These suggested that nature connectedness fully mediated the relationship between direct nature contact and expressive suppression (indirect effect = − 0.016, SE = 0.004, 95% CI: [− 0.026, − 0.008]). The mediation effects account for 47.06% of the total effect of direct nature contact on expressive suppression.

The findings illustrate that after controlling for gender, age, and education level, nature connectedness mediates the relationship between direct contact and cognitive reappraisal or expressive suppression. Thus, Hypotheses 1a and 1b were supported.

### Testing for mediation effects between indirect nature contact and emotion regulation strategies

Table 3 presents the results of the mediation analyses with indirect nature contact as the predictor of mediation model. First, after controlling for covariates, as seen in Model 1a, indirect nature contact was positively associated with cognitive reappraisal, *β* = 0.072, SE = 0.017, *p* < 0.001, 95% CI: [0.039, 0.106]. Second, as shown in Model 2, indirect nature contact was positively associated with nature connectedness, *β* = 0.022, SE = 0.009, *p* = *0.014*, 95% CI: [0.005, 0.040]. Third, as seen in Model 3a, the association between indirect nature contact and cognitive reappraisal was significant, *β* = 0.055, SE = 0.015, *p* < 0.001, 95% CI: [0.025, 0.086]; and the association between nature connectedness and cognitive reappraisal was significant as well, *β* = 0.765, SE = 0.037, *p* < 0.001, 95% CI: [0.692, 0.839]. The results showed that nature connectedness partially mediated the relationship between indirect nature contact and cognitive reappraisal (indirect effect = 0.017, SE = 0.007, 95% CI: [0.004, 0.031]). The mediation effect account for 23.61% of the total effect of indirect nature contact on cognitive reappraisal. The findings reveal that after controlling for gender, age, and education level, nature connectedness mediates the relationship between indirect nature contact and cognitive reappraisal.

**Table 3:** Testing the mediating effects of nature connectedness with indirect nature contact as the predictor.

Predictors	Model 1a (Cognitive reappraisal)		Model 1b (Expressive suppression)		Model 2 (Nature connectedness)		Model 3a (Cognitive reappraisal)		Model 3b (Expressive suppression)	
	*β*	*t*	*β*	*t*	*β*	*t*	*β*	*t*	*β*	*t*
Gender (ref. female)	0.010	0.289	0.207	3.347**	− 0.085	− 4.614***	0.075	2.369*	0.174	2.818**
Age	− 0.001	− 0.114	− 0.041	− 3.720***	− 0.002	− 0.497	0.001	0.097	− 0.042	− 3.801***
Education level (ref. undergraduate students)										
High school degree or below	0.203	1.498	0.394	1.631	− 0.092	− 1.268	0.273	2.208*	0.358	1.493
Undergraduate or college degree	0.035	0.553	0.083	0.732	− 0.006	− 0.171	0.040	0.683	0.081	0.717
Master’s degree or above	− 0.006	− 0.087	− 0.202	− 1.587	− 0.047	− 1.231	0.030	0.456	− 0.221	− 1.740
Indirect contact	0.072	4.278***	− 0.004	− 0.139	0.022	2.460*	0.055	3.579***	0.004	0.146
Nature connectedness							0.765	20.459***	− 0.386	− 5.311***
*R*^2^	0.014		0.023		0.014		0.179		0.036	
*F*	5.080***		8.066***		4.926***		65.019***		11.034***	

***p < .001, **p < .01, *p < .05; Models 1a, 2, and 3a show the mediating effect of nature connectedness between indirect contact and cognitive reappraisal; Models 1b, 2, and 3b show the mediating effect of nature connectedness between indirect contact and expressive suppression.

As shown in Model 1b of Table 3, indirect nature contact was not associated with expressive suppression, *β* = − 0.004, SE = 0.030, *p* = 0.889, 95% CI: [− 0.063, 0.055]. As shown in Model 3b, the association between indirect nature contact and expressive suppression was not significant, *β* = 0.004, SE = 0.030, *p* = 0.884, 95% CI: [− 0.054, 0.063]; but the association between nature connectedness and expressive suppression was significant, *β* = − 0.386, SE = 0.073, *p* < 0.001, 95% CI: [− 0.528, − 0.243]. The results showed that nature connectedness fully mediated the relationship between indirect nature contact and expressive suppression (indirect effect = − 0.009, SE = 0.004, 95% CI: [− 0.017, − 0.002]).

The findings illustrate that after controlling for gender, age, and education level, nature connectedness mediates the relationship between indirect contact and cognitive reappraisal or expressive suppression. Thus, Hypotheses 1a and 1b were supported.

### Moderated mediation analyses

Table 4 displays the results of the moderated mediation tests with direct or indirect nature contact as the predictor. After controlling for the covariates, Model 2a shows that the interaction between direct nature contact and engagement with natural beauty predicted cognitive reappraisal, *β* = 0.044, SE = 0.015,* p* = 0.004, 95% CI: [0.014, 0.073]. Model 3 shows that the interaction between indirect nature contact and engagement with natural beauty predicted nature connectedness, *β* = 0.028, SE = 0.008,* p* < 0.001, 95% CI: [0.012, 0.044]. Models 4a and 4b show that the interaction between indirect nature contact and engagement with natural beauty predicted cognitive reappraisal and expressive suppression, *β* = 0.038, SE = 0.015,* p* = 0.008, 95% CI: [0.010, 0.067]; and *β* = 0.079, SE = 0.030,* p* = 0.008, 95% CI: [0.021, 0.137], respectively. Thus, Hypothesis 2 was supported.

**Table 4:** Testing the moderated mediation effect with direct or indirect nature contact as the predictor.

Predictors	Model 1 (Nature connectedness)		Model 2a (Cognitive reappraisal)		Model 2b (Expressive suppression)		Model 3 (Nature connectedness)		Model 4a (Cognitive reappraisal)		Model 4b (Expressive suppression)	
	*β*	*t*	*β*	*t*	*β*	*t*	*β*	*t*	*β*	*t*	*β*	*t*
Gender (ref. female)	− 0.078	− 4.695***	0.066	2.199*	0.179	2.918**	− 0.072	− 4.334***	0.071	2.363*	0.172	2.790**
Age	− 0.005	− 1.548	− 0.003	− 0.604	− 0.043	− 3.934***	− 0.004	− 1.341	− 0.003	− 0.617	− 0.044	− 4.029***
Education level (ref. undergraduate students)												
High school degree or below	− 0.032	− 0.483	0.341	2.931**	0.386	1.614	− 0.012	− 0.177	0.362	3.091**	0.440	1.832
Undergraduate or college degree	0.038	1.258	0.118	2.168*	0.103	0.924	0.041	1.328	0.106	1.919	0.111	0.985
Master’s degree or above	− 0.022	− 0.638	0.057	0.924	− 0.215	− 1.702	− 0.025	− 0.713	0.049	0.802	− 0.209	− 1.656
Direct nature contact	0.018	2.356*	0.044	3.207**	− 0.027	− 0.979						
Indirect nature contact							− 0.003	− 0.363	0.025	1.694	− 0.010	− 0.338
Engagement with natural beauty (ENB)	0.199	21.233***	0.294	15.943***	0.124	3.274**	0.205	21.964***	0.297	16.011***	0.137	3.610***
Direct nature contact × ENB	0.008	0.998	0.044	2.905**	− 0.025	− 0.819						
Indirect nature contact × ENB							0.028	3.506***	0.038	2.653**	0.079	2.661**
Nature connectedness			0.490	12.465***	− 0.502	− 6.214***			0.493	12.489***	− 0.525	− 6.505***
Nature connectedness × ENB			− 0.006	− 0.166	− 0.067	− 0.961			0.000	0.006	− 0.076	− 1.097
*R*^*2*^	0.197		0.272		0.042		0.200		0.269		0.044	
*F*	64.210***		78.039***		9.138***		65.058***		76.727***		9.700***	

***p < .001, **p < .01, *p < .05; Models 1, 2a, and 2b show the moderating effect of ENB with direct nature contact as a predictor; Models 3, 4a, and 4b show the moderating effect of ENB with indirect nature contact as a predictor.

**Figure 2:**
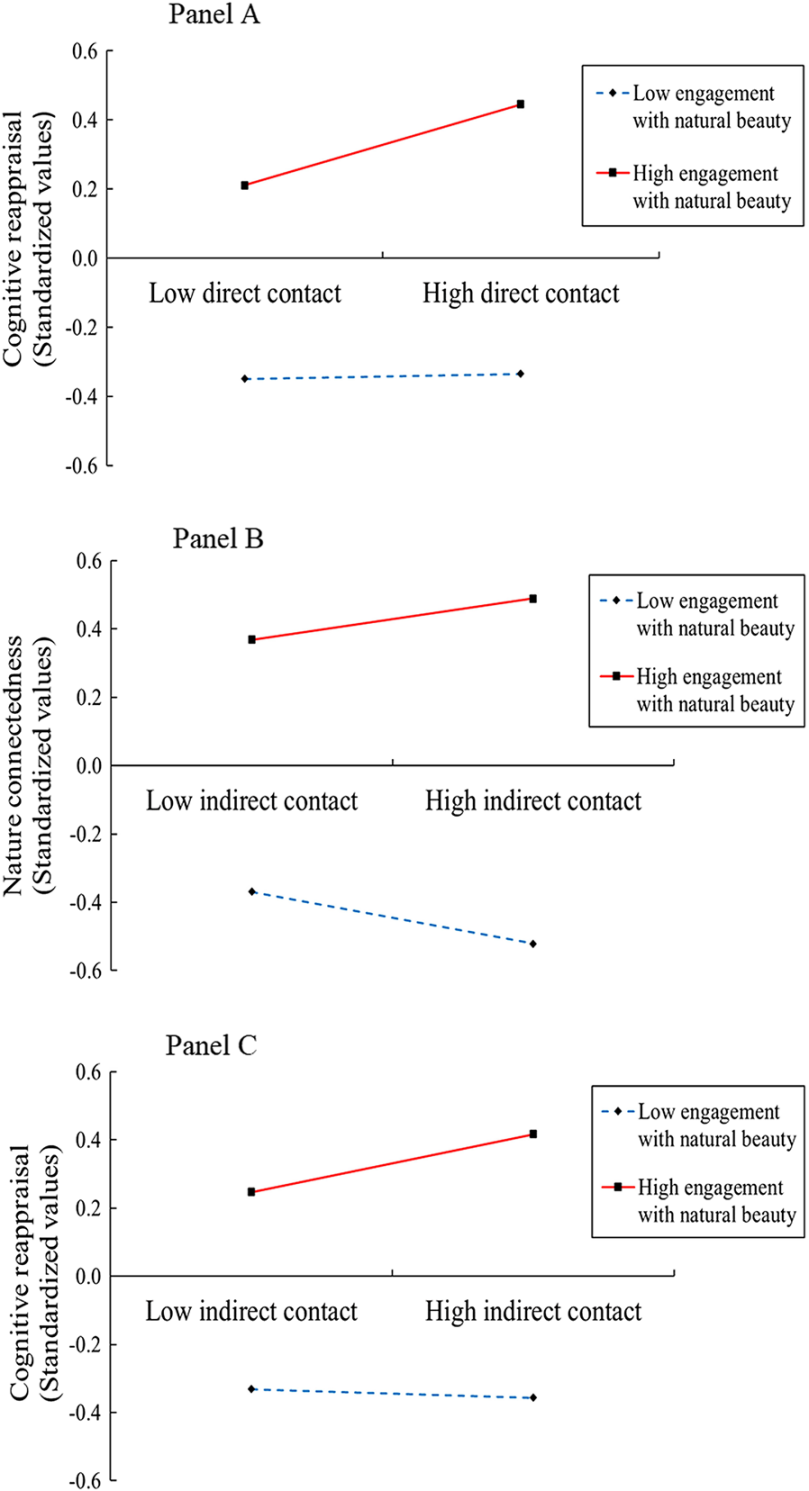
Interaction between direct/indirect nature contact and engagement with natural beauty on nature connectedness or cognitive reappraisal. Functions are graphed for two levels of engagement with natural beauty: 1 standard deviation above the mean and 1 standard deviation below the mean. Note that the graphs are for descriptive purposes only. All inferential analyses maintained the continuous values of nature contact and engagement with natural beauty.

To describe the results, we plotted the predicted direct/indirect nature contact against nature connectedness or cognitive reappraisal, separately for low and high levels of engagement with natural beauty (Fig. 2). Simple slope tests were conducted to further explain the significant interactions. We have the following findings. (a) Panel A of Fig. 2 shows that for young adults with higher engagement with natural beauty (1 SD above the mean), direct nature contact significantly predicted cognitive reappraisal, *b*_simple_ = 0.082, *p* < 0.001. However, for young adults with lower engagement with natural beauty (1 SD below the mean), the relationship between direct nature contact and cognitive reappraisal became non-significant, *b*_simple_ = 0.005, *p* = 0.791. (b) As shown in Panel B of Fig. 2, for young adults with higher engagement with natural beauty (1 SD above the mean), indirect nature contact significantly predicted nature connectedness,* b*_simple_ = 0.022, *p* = 0.038; while for young adults with lower engagement with natural beauty (1 SD below the mean), indirect nature contact significantly predicted nature connectedness as well but in an opposite direction, *b*_simple_ = − 0.028, *p* = 0.012.

(c) Panel C of Fig. 2 shows that for young adults with higher engagement with natural beauty (1 SD above the mean), indirect nature contact significantly predicted cognitive reappraisal,* b*_simple_ = 0.059, *p* = 0.002. However, for young adults with lower engagement with natural beauty (1 SD below the mean), the relationship between indirect nature contact and cognitive reappraisal was non-significant, *b*_simple_ =  − 0.009, *p* = 0.654. (d) For young adults with higher engagement with natural beauty (1 SD above the mean), indirect nature contact did not predict expressive suppression,* b*_simple_ = 0.060, *p* = 0.127; and for young adults with lower engagement with natural beauty (1 SD below the mean), indirect nature contact did not predict expressive suppression either, *b*_simple_ = − 0.080, *p* = 0.051.

## Discussion

The present study aimed to investigate how nature contact was associated with emotion regulation strategies in urban young adults and further explore the underlying psychological mediation and moderation mechanism. Using the survey, we shed light on the mechanism of nature contact on emotional regulation strategies. We found direct/indirect nature contact positively predicted connectedness to nature, then enhancing cognitive reappraisal and reducing expressive suppression. Moreover, direct/indirect contact with nature positively predicted cognitive reappraisal only when individuals had a high tendency to appreciate natural beauty (see Fig. 2 Panels A and C). For individuals who had a high engagement with natural beauty, indirect nature contact positively predicted nature connectedness, whereas for individuals who had a low engagement with natural beauty, indirect nature contact negatively predicted nature connectedness (see Fig. 2 Panel B). These findings build upon the discussion of the emotional benefits of nature exposure^11^ and provide initial insight into one possible process of nature contact on emotion regulation strategies, illuminating the mediating role of nature connectedness and the moderating role of engagement with natural beauty.

Engagement with natural beauty shapes young adults’ relationship with the natural environment and their adaptive way of regulating emotions. Our findings suggest that engagement with natural beauty acts as a filter in determining whether living a lifestyle of frequent nature contact is associated with adaptive emotion regulation strategy, i.e., cognitive reappraisal. The filter role of engagement with natural beauty applies to both direct and indirect contact with nature. The present findings echo those of a previous study examining the importance of increasing the general use of cognitive reappraisal to gain the potential benefit of virtual nature on well-being (as measured by subjective vitality)^51^. In addition, our findings first reveal the possible benefits and harms of virtual, indirect nature contact, which are determined by the high or low level of engagement with natural beauty. The harmful side aligns with the “nature deficit disorder” hypothesis^52,53^, positing that excessive exposure to electronic devices weakens their sense of connectedness to the natural world and adversely impacts their physical and mental well-being. Related to this hypothesis, our findings suggest that when individuals encounter nature-related content through electronic media, their nature connectedness can be augmented if they possess a highly aesthetic perception of nature. However, indirect exposure to nature through electronic means may prove detrimental to nature connectedness for individuals who fail to appreciate the beauty of nature. It is promising to apply the findings to nature-based therapeutic interventions such as meditation practices and forest bathing in virtual and actual natural environments, by highlighting the critical role of individuals’ aesthetic feelings and perceptions about nature.

According to Stress Reduction Theory, exposure to nature leads to a change in affective and cognitive states^54,55^. It also identifies certain visual aesthetic contents of natural environments that typically elicit affective reactions, such as water and vegetation^56,57^. The present study contributes to this theory by linking contact with nature, engagement with natural beauty, and emotion regulation. The degree of engagement with natural beauty can be understood in two ways. First, as explored here, individual differences exist in the degree of engagement. Even when placed in the same natural environment, some people perceive beauty more strongly than others. Second, the degree of wilderness in the natural environment is one of the objective attributes that affect the level of engagement with natural beauty. Wilder and less artificial urban natural landscapes bring more positive impacts on people’s physical and mental health, while highly artificial natural landscapes have a limited effect^58,59^. Moreover, biodiversity, as an aspect of perceived wilderness in urban landscapes, increases the well-being benefits of natural environments^60^. Therefore, the Stress Reduction Theory should be refined by taking into account the individual difference in engagement with natural beauty. To develop a better nature-based education/therapy programme, wilder, less artificial landscapes should be incorporated into urban natural environment planning.

Our findings align with previous research on the positive impact of nature contact on emotional health. However, as self-reported frequency of nature contact is only one approach to depict individuals’ contact with nature, we propose the following future directions. First, future research can examine the effects of unintentional exposure to nature, which is often assessed via an objective greenery index in neighborhoods or workplaces, such as a Normalized Difference Vegetation Index^61^. Second, different types of activities in nature and the motivations for those activities^62,63^ may modulate the effects of nature contact on emotion regulation strategies and connectedness to nature. Future research could investigate these factors to further elucidate the validity and boundary conditions of nature contact as an emotional health tool. Third, we assessed indicators of nature contact at the individual level. Future studies could incorporate a population-level perspective of how an individual contacts nature, such as examining the accessibility of infrastructure and the level of the artificiality of nature in urban greenspaces. These population-level properties may affect people’s frequency and intention to interact with nature and their aesthetic experiences in greenspace. Therefore, for urban young adults in China, contact with nature as a solution to emotional health may signify raising the frequency of their direct/indirect contact with the natural world and/or their motivation to psychologically connect with nature. It may also involve enhancing the green coverage within their urban lives and the accessibility of green infrastructure.

Two limitations should be addressed. First, the cross-sectional nature of the data restricts our ability to draw causal conclusions. Future research can implement experimental designs to manipulate the frequency and form of nature contact or utilize longitudinal designs to test the moderated mediation models. Second, it is noteworthy that the data were collected during the COVID-19 pandemic in China (in September to December 2021 and April 2022). Even though our participants reported that they were not quarantined, they might be less willing to go out (including doing activities in nature) during the pandemic. Hence, regarding the nature contact variable, participants might report a lower frequency of activities in nature and a higher frequency of watching programs than the typical situation before the pandemic. Yet even under the situation, we still find that nature contact benefits nature connectedness and adaptive emotion regulation strategy. Future research should replicate the current findings in the post-pandemic era.

## Conclusion

The present study sheds light on the psychological mechanism underlying the relationship between nature contact and emotion regulation strategies among young adults in urban China. Several noteworthy findings emerged from our investigation. First, direct/indirect nature contact predicted the employment of cognitive reappraisal and expressive suppression as emotion regulation strategies, a relationship mediated by nature connectedness. Second, engagement with natural beauty moderated the relationship between direct/indirect nature contact and cognitive reappraisal in the mediation models. The positive association between nature contact and cognitive reappraisal was only pronounced among individuals characterized by a strong rather than weak tendency for appreciating natural beauty. Third, engagement with natural beauty moderated the relationship between indirect nature contact and nature connectedness in the mediation models. While individuals exhibiting higher engagement with natural beauty showed a positive association between indirect nature contact and nature connectedness, this association became negative for those with a lower engagement with natural beauty. Our findings suggest that young adults in urban areas can gain emotional benefits derived from a profound affinity with nature through living a lifestyle of frequent contact with real nature and through increasing their tendency to appreciate the beauty of nature.

## Data Availability

The data that support the findings of this study are available from the corresponding author upon reasonable request.
